# Ruptured Ectopic Pregnancy in an Accessory Horn of Uterus: A Case Report

**DOI:** 10.7759/cureus.6436

**Published:** 2019-12-21

**Authors:** Zainab Abbasi, Sajan Das, Usha Thapa, Sarthak Aryal, Sumaira Mughal

**Affiliations:** 1 Internal Medicine, Liaquat University of Medical and Health Sciences, Jamshoro, PAK; 2 Emergency Medicine, Sindh Government Lyari General Hospital, Karachi, PAK; 3 Internal Medicine, Chongqing Medical University, Chongqing, CHN; 4 Internal Medicine, Peoples' Friendship University of Russia, Moscow, RUS; 5 Emergency Medicine, Civil Hospital Karachi, Karachi, PAK

**Keywords:** mullerian duct anomalies, accessory horn of uterus, ectopic pregnancy, hemoperitoneum

## Abstract

A unicornuate uterus with an accessory horn is one of the rarest congenital uterine anomalies; hence, the possibility of ectopic pregnancy in the accessory uterine horn is highly uncommon. It poses a significant risk to maternal life, as it is difficult to identify before surgery due to the severe hemoperitoneum in the event of rupture of the ectopic pregnancy. We report a case of a 20-year-old primigravida who presented to the emergency department of Civil Hospital Karachi, with sudden onset of generalized abdominal pain, vomiting, and dizziness at 17 weeks of gestation. Emergency ultrasonography of the abdomen showed extensive echogenic fluid, which was considerably obscuring the view. An empty uterus was seen with a complex cystic mass on its right side separate from the ovary. A ruptured ectopic pregnancy was suspected, and hence, the patient was immediately shifted to the operating room. Emergency laparotomy was done which then showed ruptured ectopic pregnancy with a viable fetus in a right-sided rudimentary horn of the uterus. The horn was excised. The patient recovered well, and the postoperative course was uneventful.

## Introduction

Congenital uterine anomalies originate in the instance of defects in development, fusion, or failure of female reproductive tract septum absorption during embryogenesis. The prevalence of congenital uterine anomalies in the infertile population is estimated to be 16.7%, which is slightly higher than its prevalence in the general population, which is 7.3% [1]. Unicornuate uterus with an accessory horn is an unusual congenital uterine anomaly that develops when one of the Müllerian ducts develops incompletely resulting in a partial fusion with the duct of the opposite side. The final result is the development of an accessory horn which may have a functional cavity, or it may simply be a small solid mass of the uterine muscle with no functional endometrium [2]. The majority of the cases remain undiagnosed unless a pregnancy develops in the accessory horn, and most of these patients present when the rupture has already occurred. A diagnosis of this anomaly is usually confirmed intraoperatively. Here we report a case of a patient with ruptured ectopic pregnancy in the accessory horn of uterus who presented to our tertiary care center.

## Case presentation

**Figure 1:**
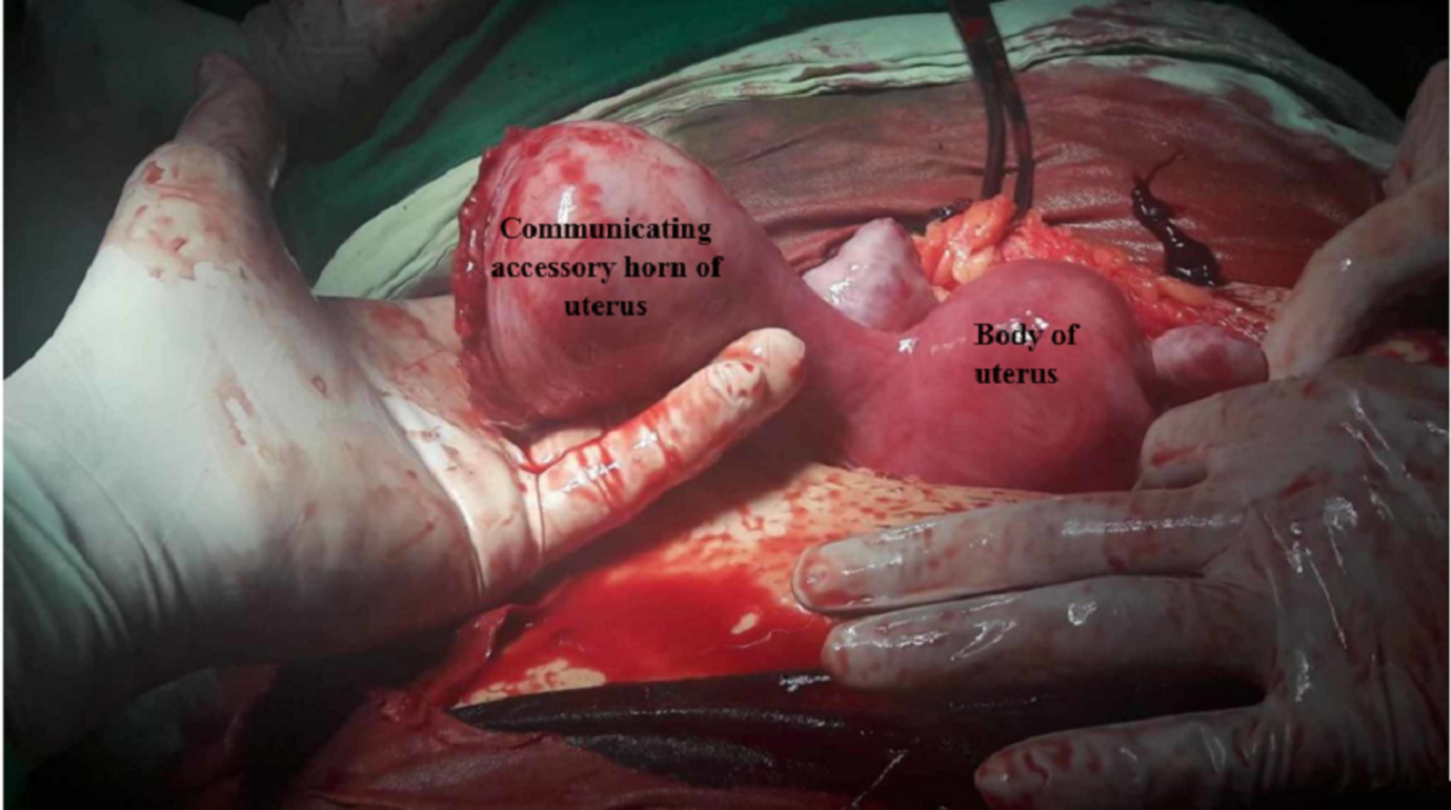
Accessory horn of uterus still attached to the main horn.

A 20-year-old woman presented to the emergency department of Civil Hospital Karachi, with sudden onset of generalized abdominal pain, vomiting, dizziness and an episode of syncope for two hours. She had amenorrhea of 17 weeks. There was no history of vaginal bleeding. She took a home pregnancy test two months ago and was positive, but she had not undergone any further testing to confirm. Her pulse was 125-bpm and her blood pressure was 50/30 mmHg. On a general physical exam, she was conscious but profoundly pale. Abdominal examination showed distention with signs of peritoneal irritation including a markedly tense abdomen with generalized tenderness on palpation. On vaginal examination, cervical os was closed however forniceal fullness was felt and cervical motion tenderness was positive. An emergent transabdominal ultrasound was done in the emergency department that showed an abnormal contour of the uterine fundus with fetal cardiac activity in the right fundal region, a large amount of fluid in the peritoneum obscuring the view. A provisional diagnosis of hypovolemic shock secondary to ruptured ectopic pregnancy was made. Blood workup, as well as cross-match, was sent. Two wide bore cannulas were inserted, I/V fluids were started and the patient was prepared for emergency exploratory laparotomy due to acute presentation. The uterus was approached via Pfannenstiel incision. Intraoperatively there was hemoperitoneum of approximately 1000 c.c. and unexpectedly, an accessory horn measuring approximately 6 x 6 cm was found arising from the right fundal region of the uterus (Figure 1).

**Figure 2:**
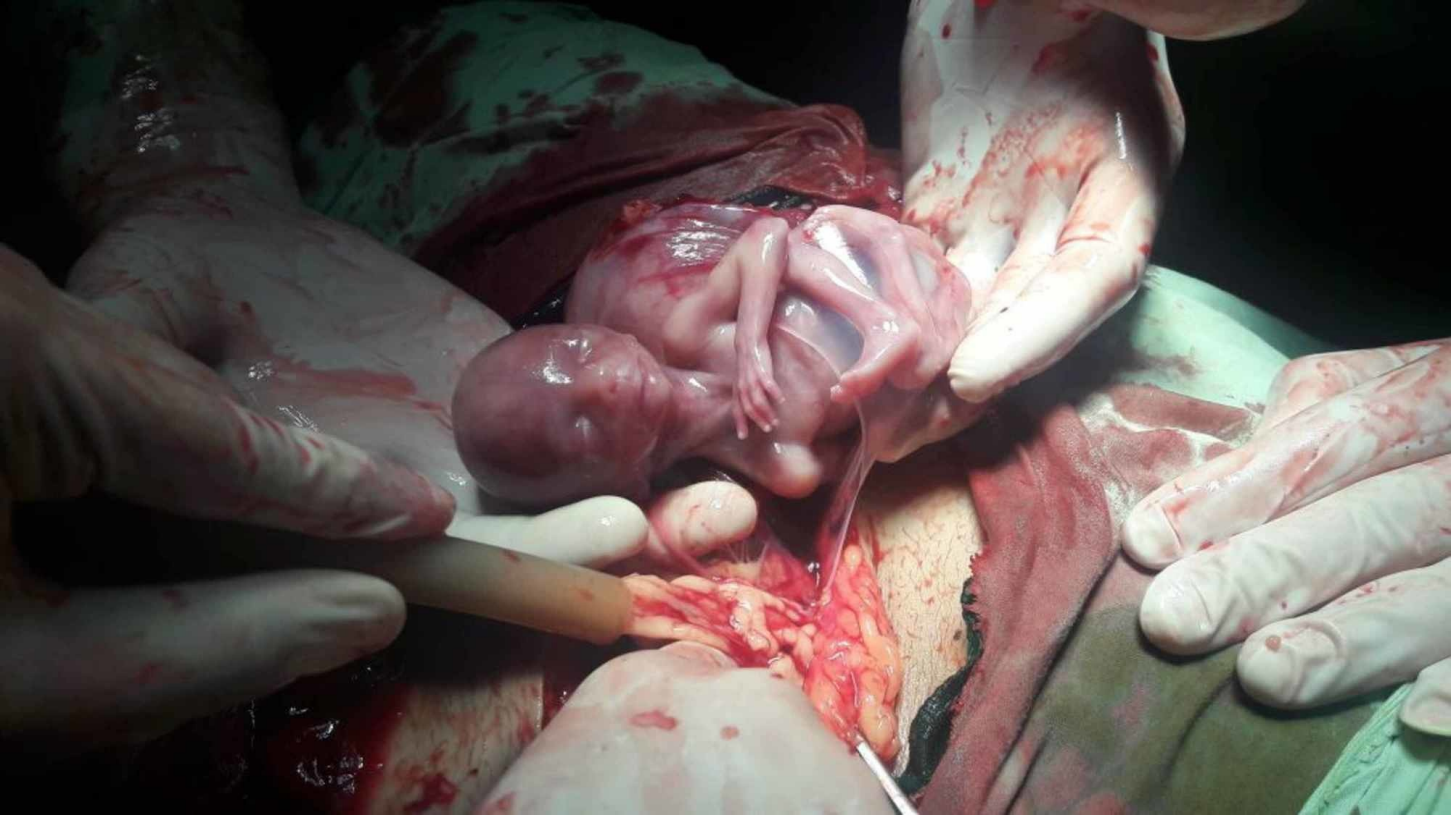
A 17-week-old fetus in a gestational sac being removed from the peritoneal cavity, partially covered by the ruptured amniotic sac.

**Figure 3:**
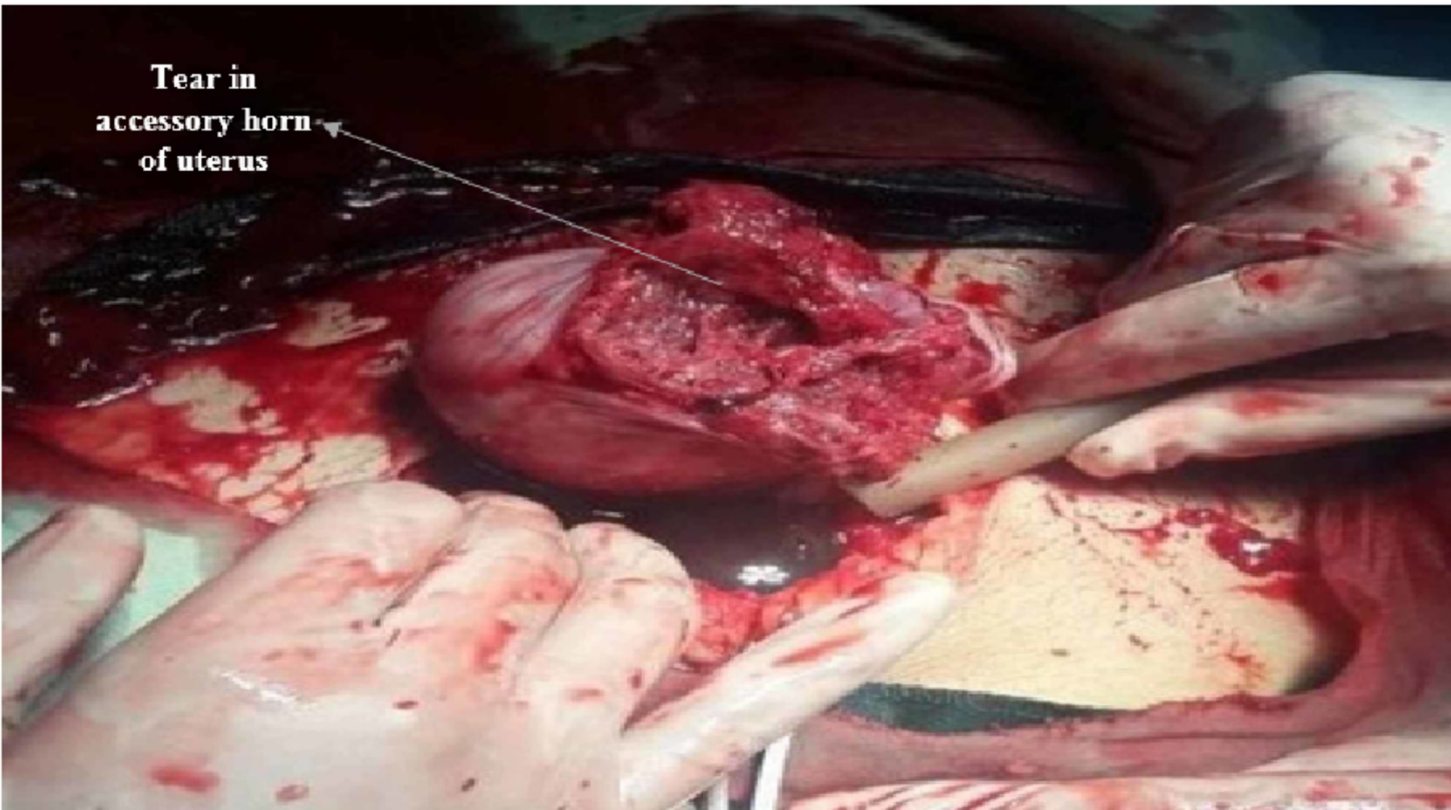
Accessory horn of uterus demonstrating a large posterior tear.

The right fallopian tube was found arising from this accessory horn, instead of the uterus itself. A ruptured gestational sac was found partially expelling the fetus in the peritoneal cavity (Figure 2) from the ruptured segment through a large posteriorly located tear (Figure 3).

**Figure 4:**
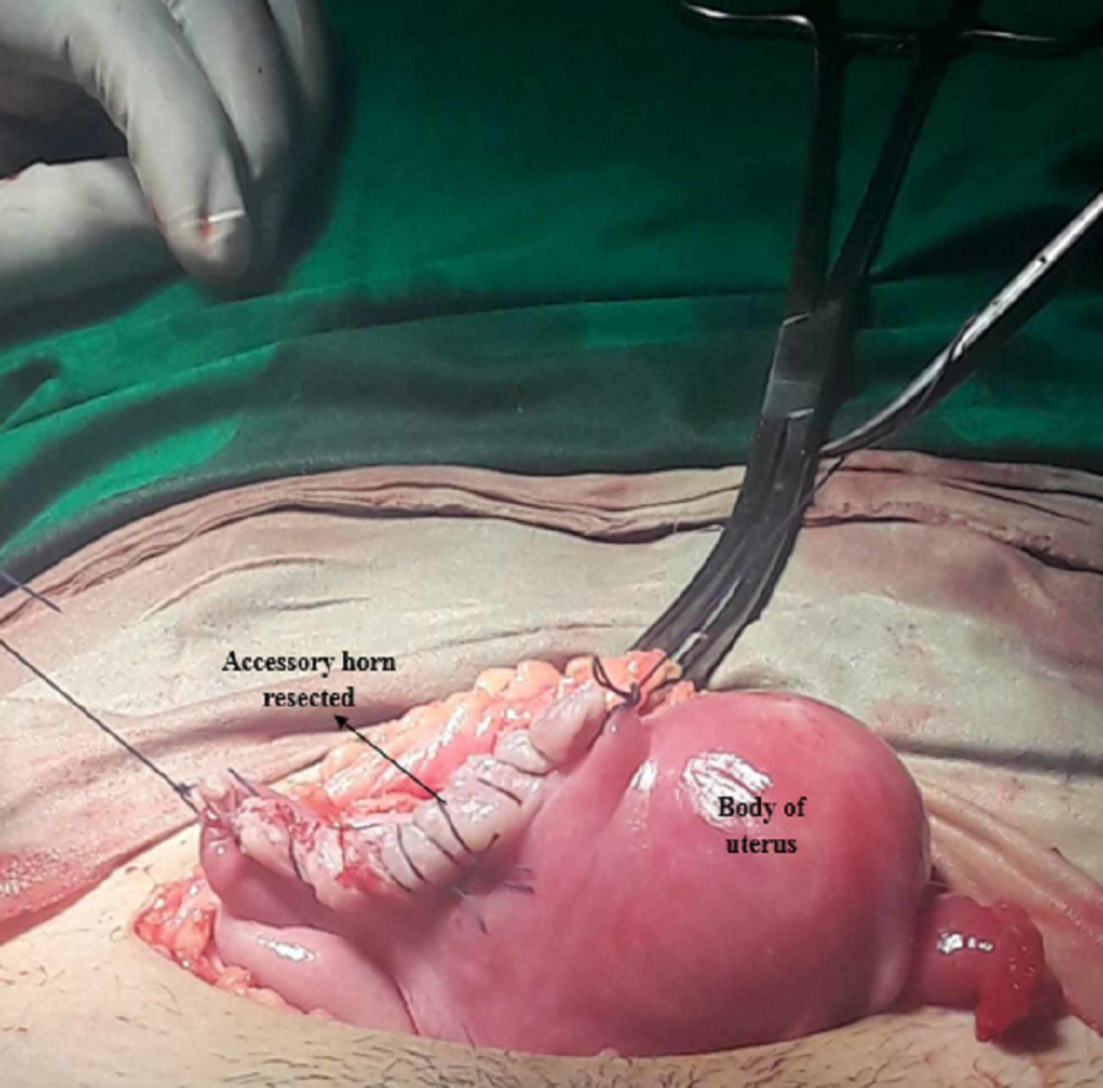
Accessory horn resected with right salpingectomy.

The umbilical cord was found attached to the endometrium in the accessory horn. The gestational sac was removed and peritoneal wash was done. The accessory horn was resected along with the right salpingectomy and hemostasis secured (Figure 4).

The left fallopian tube and both ovaries were conserved. Postoperatively the patient's course was uneventful and she was discharged on the 6th day. The resected segment was sent for histopathologic evaluation, which confirmed the diagnosis of the accessory horn of the uterus. The patient was counseled about contraception and was seen at the clinic two weeks postoperatively and had no complications.

## Discussion

Approximately 95% of the ectopic pregnancies occur in the fallopian tubes [3]. Hence ectopic pregnancy in an accessory horn of uterus that gives rise to the ipsilateral fallopian tube is a rare clinical entity. The first case of ectopic pregnancy in a rudimentary horn of uterus was published in the year 1669 by Vassal and Mauriceau [4]. Ectopic pregnancy rupture usually presents with symptoms of abdominal pain, dizziness, and syncope [5]. The uterus being a highly muscular organ has an abundant blood supply, hence the rupture of an accessory horn of uterus could lead to a life-threatening hemorrhage. The pre-rupture diagnosis still remains a challenge especially in women who do not undergo pre-pregnancy or early pregnancy diagnostic workup. Different imaging modalities could be used for early diagnosis of the accessory horn of uterus including transvaginal ultrasound, three-dimensional ultrasonography or MRI scans [6,7]. Early management of ectopic pregnancy rupture also reduces the risk of future complications like intraperitoneal adhesions [8]. In the present case, the patient presented in a clinical emergency of hypovolemic shock and signs of peritoneal irritation and hence the diagnostic measures adopted were symptoms and ultrasonography. Hence patients attending obstetric and gynecologic clinics must be educated regarding the significance of prenatal and early intra-natal imaging for confirmation of not only a viable pregnancy but also the location of that pregnancy and ruling out uterine anomalies.

## Conclusions

We present a case of a primigravida woman who presented to the emergency department with hypovolemic shock and signs of peritoneal irritation with a history of amenorrhea. Rather than being a classical case of tubal ectopic pregnancy rupture which was suspected preoperatively, she was found to have a pregnancy in an accessory horn of uterus that ruptured leading to a surgical emergency. This case highlights the importance of prenatal and early intra-natal imaging modalities for prompt diagnosis of Mullerian duct anomalies so that catastrophic outcomes could be prevented in the event of a rupture.
